# Psychiatrische Symptome der Huntington-Krankheit

**DOI:** 10.1007/s00115-024-01728-z

**Published:** 2024-08-30

**Authors:** Alzbeta Mühlbäck, Rainer Hoffmann, Nicolo Gabriele Pozzi, Martin Marziniak, Peter Brieger, Matthias Dose, Josef Priller

**Affiliations:** 1 Huntington-Zentrum-Süd, kbo-Isar-Amper-Klinikum, Region München, Taufkirchen (Vils), Deutschland; 2 Klinik und Poliklinik für Psychiatrie und Psychotherapie, Klinikum rechts der Isar, School of Medicine and Health, TU München, Ismaninger Str. 22, 81675 München, Deutschland; 3 Neurologische Klinik und Poliklinik, Universitätsklinikum Würzburg, Würzburg, Deutschland; 4 Klinik für Neurologie und Intensivmedizin, kbo-Isar-Amper-Klinikum, Region München, Akademisches Lehrkrankenhaus der LMU München, Haar, Deutschland; 5 kbo-Isar-Amper-Klinikum, Region München, Akademisches Lehrkrankenhaus der LMU München, Haar, Deutschland; 6 Deutsches Zentrum für Psychische Gesundheit (DZPG), Standort München, München, Deutschland; 7 Universität Edinburgh und UK DRI, Edinburgh, Großbritannien; 8 Neuropsychiatrie und Labor für Molekulare Psychiatrie, Charité-Universitätsmedizin Berlin, Berlin, Deutschland; 9 DZNE, Berlin, Deutschland

**Keywords:** Verhaltensauffälligkeiten, Psychische Symptome, Pharmakologische Therapie, Nichtpharmakologische Therapie, Psychotherapie, Behavioral symptoms, Mental health, Pharmacological therapy, Nonpharmacological therapy, Psychotherapy

## Abstract

Die Huntington-Krankheit (HK) ist eine autosomal-dominante Erbkrankheit, die zu motorischen, kognitiven und psychiatrischen Symptomen führt. Die Diagnose kann durch den molekulargenetischen Nachweis einer verlängerten CAG-Wiederholung im *Huntingtin*-Gen gesichert werden. Psychische und Verhaltenssymptome sind bei der HK häufig und können Jahre vor den motorischen Symptomen auftreten. Zu den psychiatrischen Symptomen gehören Apathie, Depression, Angst, Zwangssymptome und in einigen Fällen Psychosen und Aggression. Diese können aktuell nur symptomatisch behandelt werden, da sich krankheitsmodifizierende Therapieansätze bei der HK noch in der Erprobung befinden. Die derzeitige klinische Praxis basiert auf Expertenmeinungen sowie Erfahrung mit der Behandlung ähnlicher Symptome bei anderen neurologischen und psychiatrischen Krankheiten. In diesem Artikel geben wir einen Überblick über die komplexen psychischen Manifestationen der HK, die diagnostischen Möglichkeiten und die etablierten pharmakologischen und nichtpharmakologischen Behandlungsansätze.

## Abstract (en)

Huntington’s disease (HD) is an autosomal dominant inherited disease, which leads to motor, cognitive and psychiatric symptoms. The diagnosis can be confirmed by genetic testing for extended CAG repeats in the *Huntingtin* gene. Mental and behavioral symptoms are common in HD and can appear several years before the onset of motor symptoms. The psychiatric symptoms include apathy, depression, anxiety, obsessive-compulsive symptoms and, in some cases, psychoses and aggression. These are currently restricted to symptomatic treatment as disease-modifying treatment approaches are still under investigation. The current clinical practice is based on expert opinions as well as experience with the treatment of similar symptoms in other neurological and mental health diseases. This article provides an overview of the complex psychiatric manifestations of HD, the diagnostic options and the established pharmacological and nonpharmacological treatment approaches.

## Lernziele

Nach Lektüre dieses Beitrags …

- verstehen Sie die Grundlagen der Huntington-Krankheit und ihre klinischen Manifestationen,
- wissen Sie, welche psychiatrischen Symptome sich bei der Huntington-Krankheit manifestieren können,
- kennen Sie pharmakologische sowie nichtpharmakologische Ansätze zur Behandlung der Huntington-Krankheit,
- sind Sie mit den ethischen Aspekten vertraut, die bei der Betreuung von Personen mit Huntington-Krankheit auftreten können.

## Einführung

Die Entdeckung des die Huntington-Krankheit (HK) verursachenden Gens im Jahr 1993 und die damit verbundene Möglichkeit, gezielt nach innovativen Therapien zu suchen, haben in den letzten Jahren zu einer Reihe klinischer Studien geführt, die jedoch bisher keinen krankheitsmodifizierenden Therapieansatz hervorgebracht haben. Neben den motorischen und kognitiven Symptomen der HK führen vor allem **psychische Symptome**

psychische Symptome

und Verhaltensänderungen zu Leistungseinbußen im Alltag und schränken die Lebensqualität der Betroffenen erheblich ein. Im Folgenden sollen die psychiatrischen Symptome der HK und ihre **Behandlungsmöglichkeiten**

Behandlungsmöglichkeiten

dargestellt werden.

### Fallbeispiel

Eine 38-jährige Patientin stellt sich bei einem niedergelassenen Arzt vor. Sie berichtet, dass sie seit einigen Monaten Konzentrationsschwierigkeiten und vermehrt Konfliktsituationen mit Kollegen am Arbeitsplatz erlebe, da sie ihre Aufgaben nicht mehr so gut erfüllen könne. Dies alles habe sich schleichend entwickelt. Sie sei reizbar und aufbrausend geworden, was zu vielen Problemen in der Familie führe. Auch mit dem Haushalt komme sie nicht mehr zurecht. Sie schlafe nachts sehr schlecht und habe 7 kg abgenommen. Ihr Partner habe sie auf leichte Zuckungen der Finger aufmerksam gemacht. Ihre Mutter sei gesund und helfe ihr im Alltag. Ihr Vater sei vor vielen Jahren bei einem Autounfall ums Leben gekommen. In den letzten Jahren habe er unter Depressionen gelitten. Der Großvater väterlicherseits sei an Demenz gestorben, habe auch ein unsicheres Gangbild gehabt, die Großmutter sei mit 75 Jahren an einem Herzinfarkt gestorben. Die Großeltern mütterlicherseits seien über 80 Jahre alt und altersentsprechend gesund. Weitere Erkrankungen in der Familie sind nicht bekannt.

## Hintergrund

Die Huntington-Krankheit (HK) ist eine neurodegenerative Erkrankung, die durch eine Trias von motorischen Symptomen (vor allem Bewegungsstörungen), kognitiven Beeinträchtigungen und psychiatrischen Symptomen gekennzeichnet ist. Die Krankheit ist nach **George Huntington**

George Huntington

benannt, der als praktizierender Hausarzt die Krankheit und ihre Erblichkeit in mehreren Familien auf Long Island, New York, beschrieb [1].

Der HK liegt eine Mutation des **Huntingtin-Gens**

Huntingtin-Gens

(*HTT*) zugrunde, die durch übermäßige Wiederholung (**„repeats“**

**Abb. 1:**
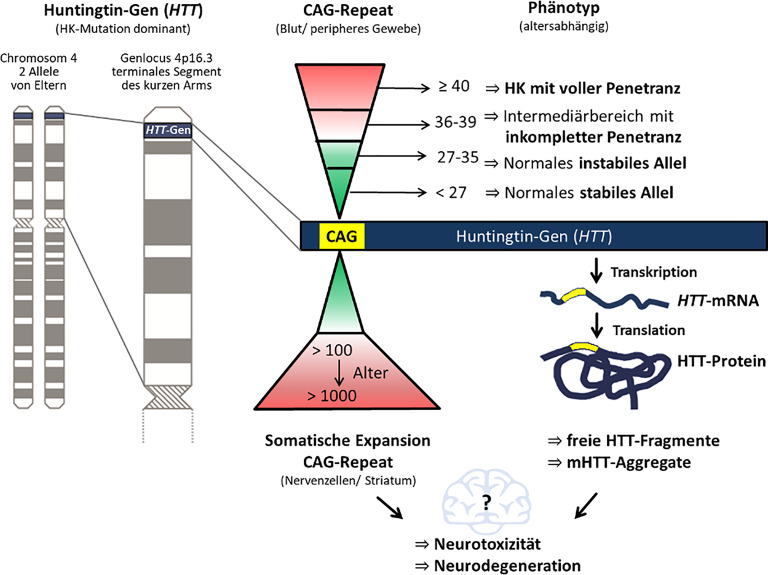
Pathophysiologie der Huntington-Krankheit (*HK*)

„repeats“)

dreier Basen (CAG) im Exon 1 auf Chromosom 4 (4p16.3) verursacht wird und u. a. zur Bildung des mutierten Huntingtin-Proteins (mHTT) führt, das für die Pathologie und Toxizität mitverantwortlich ist ([2]; Abb. 1). Die Länge der **CAG-Wiederholungen**

CAG-Wiederholungen

bestimmt, ob eine Person an HK erkrankt [3]. Die Anzahl der CAGs in der Allgemeinbevölkerung liegt im Bereich von 6 bis 35 CAG-Wiederholungen, bei ≥ 40 CAG-Wiederholungen zeigt die Mutation volle Penetranz und löst einen Krankheitsprozess aus, der unvermeidlich zu den Symptomen der HK führt [3]. Im Bereich zwischen 36 und 39 CAGs ist eine inkomplette Penetranz bekannt, d. h. die Betroffenen erkranken zu Lebzeiten nicht oder zeigen erst spät erste Symptome [3].

CAG-Wiederholungen zwischen 27 und 35 CAGs (auch als **intermediäre Allele**

intermediäre Allele

bezeichnet) sind in der Regel nicht mit Krankheitssymptomen assoziiert, aber die Möglichkeit der Expansion der CAG-Repeats stellt ein erhöhtes Erkrankungsrisiko für die Nachkommen dar [3, 4]. Das Vorhandensein dieser intermediären Allele ist einer der möglichen Gründe, warum Krankheitsfälle beobachtet werden, obwohl bisher niemand in der Familie erkrankt war [4]. Es besteht eine inverse Beziehung zwischen der Zahl der CAG-Wiederholungen und dem **Erkrankungsalter**

Erkrankungsalter,

d. h. eine höhere Wiederholungszahl bedingt ein früheres Auftreten von Symptomen und ein rascheres Fortschreiten der HK [3]. Das durchschnittliche Erkrankungsalter liegt bei ca. 45 Jahren, die HK kann aber auch schon in der Kindheit oder erst im späteren Leben auftreten.

Die HK ist in allen ethnischen Gruppen endemisch, tritt aber bei Menschen **europäischer Abstammung**

europäischer Abstammung

mit 17,2 Fällen pro 100.000 häufiger auf [5]. Da immer mehr Europäer ein hohes Lebensalter erreichen und die Behandlungsmöglichkeiten besser werden, ist eine Zunahme der Prävalenz wahrscheinlich [4].

Der **Verlauf der HK**

Verlauf der HK

kann klinisch deskriptiv in prämanifeste und manifeste Stadien eingeteilt werden, wobei auch von einer Prodromalphase (mit subklinischer Symptomatik) in den Jahren vor Ausbruch der HK gesprochen wird [6].

Darüber hinaus wurde zu Forschungszwecken eine neue **Klassifikation**

Klassifikation

– das Huntington’s Disease Integrated Staging System (HD-ISS) – eingeführt, die Personen von Geburt an charakterisiert, beginnend mit **Stadium 0**

Stadium 0

(d. h. Personen mit der genetischen Mutation für die Huntington-Krankheit ohne nachweisbare Pathologie), dem Fortschreiten der Huntington-Krankheit anhand messbarer Indikatoren (z. B. Magnetresonanztomographie, MRT) der zugrunde liegenden Pathologie (**Stadium 1**

Stadium 1),

einem nachweisbaren klinischen Phänotyp (**Stadium 2**

Stadium 2)

und schließlich dem funktionellen Abbau (**Stadium 3**

Stadium 3;

[7]). Es wird erwartet, dass sich diese Klassifikation in der klinischen Praxis durchsetzt.

Die Anlageträger der Mutation im *HTT*-Gen, umgangssprachlich auch **„Genträger“**

„Genträger“

genannt, unterscheiden sich klinisch und funktionell in den ersten Lebensjahren typischerweise nicht von Personen ohne Mutation im *HTT*-Gen [6]. Erst im Laufe des Lebens entwickeln Mutationsträger kognitive Beeinträchtigungen, psychiatrische Symptome, Verhaltensauffälligkeiten und motorische Symptome (z. B. unwillkürliche choreatische Bewegungen), die zu einer zunehmenden Beeinträchtigung im Alltag, Pflegebedürftigkeit und zum Tod führen [6, 8].

Die **motorischen Symptome**

motorischen Symptome

der HK lassen sich in zwei Hauptkomponenten unterteilen:

- die unwillkürlichen Bewegungsstörungen (z. B. Chorea) und
- die Beeinträchtigung der willkürlichen Bewegungen mit Koordinationsstörungen und Bradykinesie, die typischerweise mit Rigor, Dysarthrie, Dysphagie und Gangstörungen einhergehen, sowie Dystonien in den Spätstadien der Erkrankung [6].

Eine Ausnahme bilden die juvenilen Formen der Erkrankung, die bereits im Frühstadium mit bradykinetischen und dystonen Symptomen einhergehen [6].

Die **kognitive Beeinträchtigung**

kognitive Beeinträchtigung

beginnt sehr früh und schreitet wie die motorischen Symptome allmählich voran [9]. Die Merkmale der kognitiven Beeinträchtigung ähneln denen anderer **striatal-subkortikaler Hirnerkrankungen**

striatal-subkortikaler Hirnerkrankungen

(z. B. Parkinson-Krankheit). HK-Patienten können Probleme mit der Aufmerksamkeit, der kognitiven Flexibilität, der Planung, dem Erkennen von Emotionen und eine psychomotorische Verlangsamung haben [10]. Die Sprache bleibt bei der HK meist lange gut erhalten, aber es entwickelt sich eine zunehmende Dysarthrie, im Stadium der Demenz häufig auch Wortfindungsstörungen [6].

Neben den kognitiven Beeinträchtigungen können **psychiatrische Symptome**

psychiatrische Symptome

auftreten, die sich als Ängste, Depressionen, Impulskontrollstörungen, Antriebsstörungen, Apathie, Zwänge, Unruhe und Aggressivität sowie psychotische Symptome manifestieren können [10]. Obwohl motorische Symptome als die charakteristischsten Symptome der HK angesehen werden, berichten sowohl Menschen mit HK als auch ihre Familien, dass die psychischen Symptome teilweise mehr zur **Beeinträchtigung der Lebensqualität**

Beeinträchtigung der Lebensqualität

beitragen als die motorischen Symptome.

Die HK geht auch mit einem **katabolen Zustand**

katabolen Zustand

einher, und ein fortschreitender **Gewichtsverlust**

Gewichtsverlust

ist ein gut dokumentiertes Merkmal der Krankheit, das zusammen mit Schluckstörungen zu klinischen Komplikationen wie Mangelernährung bis hin zur Kachexie führt [11, 12].

### Merke

- Die HK zeichnet sich durch eine Trias von motorisch-neurologischen, kognitiven und psychiatrischen Symptomen aus.

## Betroffene Areale bei der Huntington-Krankheit

Die Komplexität und Vielfalt der klinischen Symptome der HK erschließt sich nur, wenn die Gesamtheit der beteiligten Hirnareale berücksichtigt wird. Veränderungen des Gehirns sind bereits 10 bis 15 Jahre vor dem Auftreten der ersten motorischen Symptome nachweisbar und schreiten allmählich voran [9]. Die neuronale Degeneration betrifft früh das **Striatum**

Striatum

in den Basalganglien, insbesondere die mittelgroßen dornentragenden Projektionsneurone (**„medium spiny neurons“**

„medium spiny neurons“,

MSN), breitet sich aber auf andere Strukturen des Gehirns aus [13, 14]. Die **Basalganglien**

Basalganglien

sind an einer Vielzahl von Funktionen beteiligt, darunter willkürliche und unwillkürliche Bewegungen, prozedurales Lernen, Gewohnheitsbildung, Kognition und Emotion. Ebenso spielt auch das Muster der kortikalen Degeneration eine wichtige Rolle bei der Ausprägung des klinischen Phänotyps [13].

Die Neurodegeneration vor allem der **Pyramidenzellen**

Pyramidenzellen

des primären motorischen Kortex trägt zu den motorischen Symptomen der HK bei. Die Schädigung der Pyramidenzellen im zingulären Kortex, wo Emotionen verarbeitet werden, ist dagegen hauptsächlich mit affektiven Symptomen assoziiert [15]. Das Symptom der Apathie ist mit einer starken Abnahme der grauen Substanz innerhalb des kortikal-subkortikalen Netzwerks verbunden, wobei die **bilaterale Amygdala**

bilaterale Amygdala

und der temporale Kortex am stärksten betroffen sind [16].

Im Hypothalamus wurden Veränderungen in verschiedenen Kernen dieser Region festgestellt, z. B. im **Nucleus suprachiasmaticus**

Nucleus suprachiasmaticus,

einer Schlüsselregion für die Regulation von Schlaf und zirkadianem Rhythmus [17]. Ebenso wurde berichtet, dass die Degeneration der wichtigsten efferenten Neuronen des Kleinhirns, der **Purkinje-Zellen**

**Abb. 2:**
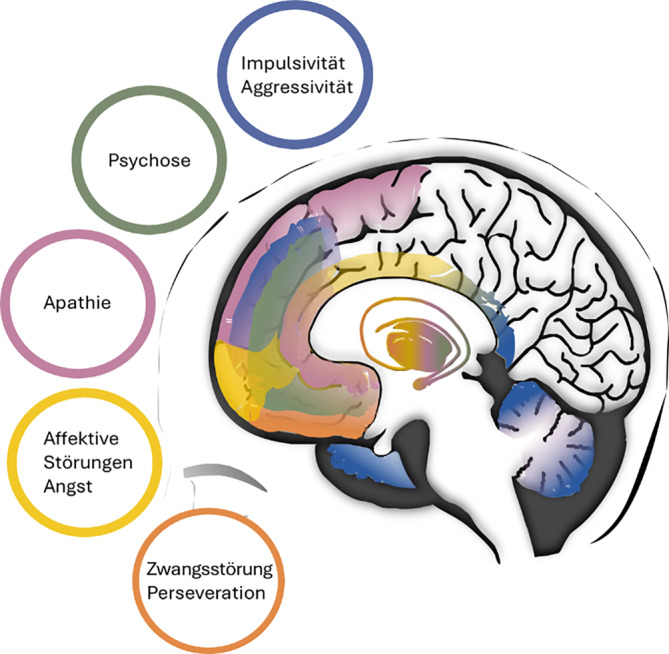
Betroffene Areale bei der Huntington-Krankheit

Purkinje-Zellen,

auch ein Merkmal der HK mit vorherrschenden motorischen Symptomen sein kann [18]. Die psychischen Symptome der HK werden mit den Stammganglien und anderen Hirnarealen in Verbindung gebracht (Abb. 2).

### Merke

- Die Vielfalt der klinischen Symptome der Huntington-Krankheit erschließt sich nur, wenn die Gesamtheit der beteiligten Hirnareale berücksichtigt wird.
- Die HK betrifft neben den Basalganglien auch andere Hirnareale wie den Kortex, die Amygdala sowie das Kleinhirn.

## Psychiatrische Symptome der Huntington-Krankheit

Die klinische Diagnose einer manifesten HK basiert aktuell auf dem Vorhandensein motorischer Symptome wie **Chorea und Bradykinesie**

Chorea und Bradykinesie

[19]. Psychische Symptome und Verhaltensauffälligkeiten können jedoch schon viele Jahre zuvor auftreten [9]. Die Prävalenz psychiatrischer Symptome liegt zwischen 33 und 76 % [9, 19]. Dazu gehören affektive Labilität, Depression, Ängste und zwangsähnliche Symptome, Reizbarkeit, Apathie und (seltener) Psychosen [20].

Die große Beobachtungsstudie „Registry“ [21], die auch an mehreren Zentren in Deutschland durchgeführt wurde, ergab, dass nur 27 % der Betroffenen keine psychiatrischen Symptome aufwiesen. Die restlichen 73 % litten unter mäßiger bis schwerer **Apathie**

Apathie

(28 %), Depression (12,7 %), Reizbarkeit und Aggressivität (13 %) sowie Zwängen und Perseverationen (13,2 %; [20]). Viele Symptome überschneiden sich und führen zu komplexen psychiatrischen Phänomenen, wie z. B. Apathie bei gleichzeitiger **Impulskontrollstörung**

Impulskontrollstörung.

Die psychiatrischen Symptome beeinträchtigen die Autonomie und Lebensqualität der Patienten erheblich, haben massive Auswirkungen auf das soziale Leben und können stationäre Behandlungen und/oder Unterbringung in Pflegeeinrichtungen notwendig machen [19, 22]. Sie müssen daher möglichst rechtzeitig erkannt und behandelt werden [22].

Es ist zu beachten, dass sich die HK im höheren Lebensalter (unabhängig von der Zahl der CAG-Wiederholungen) häufiger motorisch, seltener psychiatrisch manifestiert [23]. Obwohl mehr als 40 % der Patienten mindestens ein psychiatrisches oder kognitives Symptom vor den motorischen Symptomen aufwiesen, wobei Depressionen am häufigsten waren, ist bei der klinischen Diagnose von **jungen Menschen**

jungen Menschen,

die durch genetische Tests als HK-Mutationsträger identifiziert wurden, Vorsicht geboten [23]. Hier sollte das Gesamtbild berücksichtigt werden und eine umfassende Diagnostik in spezialisierten Ambulanzen oder **Huntington-Zentren**

Huntington-Zentren

durchgeführt werden.

### Affektive Störungen und Suizidalität

**Depressive Symptome**

Depressive Symptome

zählen mit einer Prävalenz von 30–70 % zu den häufigsten Symptomen bei der HK [24, 25]. Sie treten in allen Stadien der HK auf und sind eng mit Suizidalität und anderen psychiatrischen Komorbiditäten verbunden. Die Pathophysiologie der Depression bei der HK ist noch nicht vollständig verstanden, aber neben reaktiven Anteilen sind bei der HK Störungen von** Neurotransmittern**

Neurotransmittern

wie Serotonin und **neurophysiologischen Regelkreisen**

neurophysiologischen Regelkreisen,

wie z. B. die Hypothalamus-Hypophysen-Nebennieren-Achse, nachweisbar [24]. Es werden Veränderungen von Verbindungen der Basalganglien, des präfrontalen Kortex sowie limbischer und paralimbischer Strukturen mit der Entstehung depressiver Symptome in Verbindung gebracht [24].

Insgesamt haben 20–30 % der Patienten mit HK **Suizidgedanken**

Suizidgedanken

und 7–10 % unternehmen einen Suizidversuch [26], im Vergleich zu 1–3 % in der Allgemeinbevölkerung [27]. Suizidgedanken bleiben oft während des gesamten Krankheitsverlaufs bestehen; Depressionen, Angstzustände oder Impulskontrollstörungen erhöhen das Risiko für suizidales Verhalten, weshalb das Vorhandensein psychischer Symptome sorgfältig überwacht werden sollte [26] und depressiven Symptomen besondere Aufmerksamkeit zu schenken ist. Es wurde festgestellt, dass nur 55 % aller Patienten mit mittelschweren bis schweren Depressionen im Zusammenhang mit HK Antidepressiva einnahmen, was auf eine **Unterbehandlung**

Unterbehandlung

hindeutet [20].

### Angststörungen

Angststörungen treten bei bis zu 50 % der Betroffenen in allen Stadien der Krankheit auf [25] und beginnen oft schon vor dem Auftreten motorischer Symptome. Neben Angstsymptomen, die durch die psychische Belastung durch das eigene Schicksal, aber auch durch die Erlebnisse in der Familie bedingt sind und in Form **konkreter Ängste**

konkreter Ängste

in Erscheinung treten, gibt es auch unspezifische und **diffuse Ängste**

diffuse Ängste,

insbesondere durch die Veränderungen z. B. der Amygdala, des Striatums und des präfrontalen Kortex [15, 28]. Störungen insbesondere des **serotonergen und dopaminergen Systems**

serotonergen und dopaminergen Systems

werden bei der Entstehung von Angst eine wesentliche Rolle beigemessen [28].

Bei der HK können sich Ängste als **Panikattacken**

Panikattacken,

generalisierte Angststörung, isolierte Phobien und soziale Phobien manifestieren [22, 29]. Zudem kann die Selbstwahrnehmung von Symptomen Ängste auslösen. Im fortgeschrittenen Stadium können in Ermangelung verbaler Kommunikationsmöglichkeiten Ängste auch als eine vermehrte Unruhe in Erscheinung treten [22].

### Antriebsstörung und Apathie

Antriebstörung sowie Apathie äußern sich in einem **Mangel an Interesse**

Mangel an Interesse

und/oder Motivation für Aktivitäten, die früher Spaß gemacht haben, sowie für das tägliche Leben und soziale Interaktionen. Es handelt sich um ein sehr häufiges neuropsychiatrisches Symptom der HK, mit zunehmender Prävalenz im Krankheitsverlauf [25, 30, 31]. Als Ursache der Apathie werden kognitive, emotionale und **autoaktivierende Defizite**

autoaktivierende Defizite

diskutiert [32, 33], die mit verschiedenen Veränderungen frontokortikaler Trakte der weißen Substanz einhergehen [34]. Die Apathie kann sich bereits im **Prodromalstadium**

Prodromalstadium

der HK, korrelierend mit einer Volumenminderung im Putamen und Kaudatum, manifestieren [35]. Bei der HK muss die Apathie von den Symptomen einer Depression unterschieden werden, was klinisch in der Regel gut möglich ist, und eine gezielte Behandlung der Symptome ermöglicht. Die Apathie korreliert direkt mit der Krankheitsprogression bei HK und ist ein Zeichen für die **kortikostriatale Beeinträchtigung**

kortikostriatale Beeinträchtigung

[31]. Apathie ist mit schlechter kognitiver Leistungsfähigkeit und einer Zunahme motorischer und psychiatrischer Beeinträchtigungen assoziiert [36].

### Zwangsähnliche Verhaltensmuster und Perseveration

Mit dem Fortschreiten der HK können zwangsähnliche Verhaltensmuster auftreten, die Ausdruck einer zunehmenden **orbitofrontalen und striatalen Dysfunktion**

orbitofrontalen und striatalen Dysfunktion

sind [37]. Eine Differenzierung zwischen Zwangsstörungen (mit Zwängen) und Perseveration (das „Haftenbleiben“ an Gedanken, aber auch „repetitives“ Verhalten), die auf Veränderungen der exekutiven Funktionen zurückzuführen sind, ist klinisch wichtig [37]. Obwohl diese Verhaltensmuster auf den ersten Blick ähnlich erscheinen (repetitives Verhalten), unterscheiden sich die möglichen Behandlungswege je nach Ursache erheblich.

Perseveration lässt sich wahrscheinlich am besten von Zwangsstörungen unterscheiden, da sie auftritt, ohne dass sich der Betroffene der Problematik bewusst ist und sie meist nicht als störend empfindet [37]. Perseverationen stellen eine **Herausforderung für die Familie**

Herausforderung für die Familie

und das Pflegepersonal dar, da der Betroffene das gleiche Verhalten (z. B. Wiederholung von Fragen, auf die Toilette gehen, rauchen, etwas trinken wollen) viele Male hintereinander, manchmal über Stunden, wiederholt. Im Gegensatz zu Perseveration treten Zwangsstörungen bei HK nicht signifikant häufiger als in der Allgemeinbevölkerung auf [38].

### Reizbarkeit, Impulsivität und Aggressivität

Es wird angenommen, dass Reizbarkeit, Impulsivität und Aggressivität bei der HK aus der komplexen Beziehung zwischen den neurobiologischen Veränderungen, die im Krankheitsverlauf auftreten, und den psychischen Reaktionen auf die subjektiv von den Betroffenen wahrgenommenen Veränderungen resultieren [39, 40, 41]. Impulsivität als **Handeln ohne Voraussicht**

Handeln ohne Voraussicht

oder Rücksicht auf mögliche Konsequenzen und Reizbarkeit als Zustand der Ungeduld und Intoleranz treten bei der HK häufig gemeinsam auf. Sie können aber auch isoliert voneinander auftreten und zu **aggressiven Ausbrüchen**

aggressiven Ausbrüchen

führen [42], in den meisten Fällen in Verbindung mit anderen neuropsychiatrischen Symptomen wie kognitive Beeinträchtigung, Apathie oder Perseveration [30].

Angehörige, Betreuer und Pflegepersonal sind in der Regel am meisten von diesen Verhaltensmustern betroffen, da sie in engem Kontakt mit den Betroffenen stehen und sie im Alltag betreuen [8]. Aggressive Episoden können durch die **geringste Provokation**

geringste Provokation

ausgelöst werden und zu wütendem oder im Extremfall gewalttätigem Verhalten führen, das stundenlang anhalten kann. Manche Erkrankte zeigen nach diesen Ausbrüchen Einsicht, sind über das Ausmaß überrascht und fühlen sich schuldig [43]. Es ist jedoch nicht ungewöhnlich, dass Erkrankte während oder nach aggressiven Ausbrüchen **uneinsichtig**

uneinsichtig

oder sich ihrer Handlungen nicht bewusst sind, was die Gesamtsituation und die Betreuung zusätzlich erschweren kann [43].

Es ist wichtig zu betonen, dass auch Hunger, Schmerzen oder andere belastende Empfindungen bei **erschwerter Kommunikation**

erschwerter Kommunikation

zu Erregung und Reizbarkeit beitragen können, was durch entsprechende Überprüfung/Untersuchung ausgeschlossen werden sollte [42].

### Psychose

Psychotische Symptome (Wahnvorstellungen, Halluzinationen) werden in der Literatur zur HK meist mit einer Prävalenz von ca. 10 % angegeben, wobei Wahnvorstellungen häufiger als Halluzinationen zu beobachten sind [25, 44]. Bei den **Halluzinationen**

Halluzinationen

überwiegen akustische Halluzinationen. Psychotische Symptome treten häufiger bei manifest erkrankten Personen auf [25]. Die **Wahnvorstellungen**

Wahnvorstellungen

können Verfolgungswahn, Eifersuchtswahn, überwertige, aber auch nihilistische Gedanken beinhalten [25, 44]. Psychotische Symptome betreffen aber auch alle anderen möglichen Inhalte wie **Zönästhesien**

Zönästhesien

wie z. B. „Kribbeln und Jucken“ als Ausdruck von „Parasitenbefall“, bis hin zu **„Folie à deux“**

„Folie à deux“,

bei der Wahnvorstellungen einer anderen Person übernommen werden [44]. Es sollte differenzialdiagnostisch eine drogen- und/oder medikamenteninduzierte psychotische Symptomatik ausgeschlossen werden [22].

Mit Zunahme der kognitiven Einschränkungen lässt die Intensität der psychotischen Symptomatik häufig nach. Psychotische Symptome sind manchmal schwer zu behandeln [45] und sind dann ein häufiger Grund für **stationäre Behandlungen**

stationäre Behandlungen

und ggf. Unterbringung in einem Pflegeheim [8].

#### Merke

- Psychiatrische Symptome können lange vor dem Auftreten typischer motorischer Symptome bei der HK auftreten und stellen eine enorme Belastung für die Betroffenen und ihre Familien dar.
- Die Patienten weisen ein erhöhtes Risiko für Suizidgedanken, Suizidversuche und Suizide auf.

## Diagnostik

### Klinische Diagnose

Ein Krankheitsbild mit **choreatischen Bewegungsstörungen,**

choreatischen Bewegungsstörungen,

kognitivem Abbau und psychiatrischen Störungen sowie (falls erfragbar) einer positiven Familienanamnese impliziert klinisch die Verdachtsdiagnose einer HK. Sie ist die häufigste Ursache „choreatischer Bewegungsstörungen“ und wird bei ca. 90 % der Betroffenen durch einen Gentest bestätigt [46]. Differenzialdiagnostisch sind andere seltene neurologische Erkrankungen in Erwägung zu ziehen, die der HK ähneln und auch als **„HK-Phänokopien“**

„HK-Phänokopien“

bezeichnet werden. Ebenso können im Rahmen von Autoimmun‑, Stoffwechsel- und neurodegenerativen Prozessen HK-ähnliche Symptome auftreten (siehe dazu [46, 47]).

Eine genaue Anamnese mit vollständiger **Familienanamnese**

Familienanamnese

ist unerlässlich, um den Krankheitsverlauf zu beschreiben und den autosomal-dominanten Erbgang zu erkennen. Die klinische Beurteilung sollte die Erhebung des psychopathologischen Befundes, die neurologische und internistische Untersuchung sowie die Erfassung von Komorbiditäten umfassen. Standardisierte **klinische Bewertungsskalen**

klinische Bewertungsskalen

(wie die Unified Huntington’s Disease Rating Scale [UHDRS]) sollten angewendet werden, da sie alle von der HK betroffenen Bereiche (motorisch, psychiatrisch und kognitiv) erfassen und zur Beurteilung des Funktionsniveaus und der Aktivitäten des täglichen Lebens sowie des Krankheitsverlaufs nützlich sind [48]. Eine **zerebrale Bildgebung**

zerebrale Bildgebung

sollte die Diagnostik der HK vervollständigen, wobei der striatale Volumenverlust das empfindlichste Maß für krankheitsbedingte Veränderungen ist [49, 50].

### Molekulargenetische Diagnostik

Die **genetische Beratung**

genetische Beratung

entsprechend dem „Gendiagnostik-Gesetz“ (GenDG) ist sowohl im Falle einer genetischen Untersuchung bei **Symptomträgern**

Symptomträgern

als auch bei einer prädiktiven Untersuchung sog. **„Risikopersonen“**

„Risikopersonen“

essenziell. Sie ist wichtig, um den Betroffenen und ihren Familien informierte Entscheidungen zu ermöglichen und die möglichen Implikationen eines Mutationsnachweises zu verstehen. In der S2k-Leitlinie Chorea/Morbus Huntington (2023; [51]) wird bei symptomatischen Patienten mit typischem klinischen Bild eine molekulargenetische Untersuchung zur Diagnosesicherung empfohlen. Gemäß GenDG [52] kann diese differenzialdiagnostische Abklärung bei symptomatischen Patienten von jedem Arzt durchgeführt werden. Bei asymptomatischen Personen hingegen ist gemäß § 10 des GenDG eine genetische Untersuchung nur durch Fachärzte für Humangenetik oder andere Ärzte zulässig, die sich durch den Erwerb einer Facharzt‑, Schwerpunkt- oder Zusatzbezeichnung für genetische Untersuchungen qualifiziert haben.

Es wird grundsätzlich empfohlen, vor einer genetischen Untersuchung und bei der Ergebnismitteilung eine genetische Beratung, ggf. auch eine **psychologische Begleitung**

psychologische Begleitung,

anzubieten. Während des Beratungsprozesses ist es unerlässlich, auf psychische Symptome, insbesondere auf Depressivität und Suizidalität, zu achten. In Einzelfällen kann es sinnvoll sein, den Prozess durch eine **ambulante Psychotherapie**

ambulante Psychotherapie

zu begleiten.

Über das GenDG hinaus haben sich die z. B. im Europäischen Huntington-Netzwerk (**EHDN**

EHDN)

zusammengeschlossenen Zentren auf Empfehlungen zur Durchführung der prädiktiven genetischen Untersuchung geeinigt [53], deren verbindliche Anwendung auch von der Deutschen Huntington-Hilfe (**DHH**

DHH

e. V.) empfohlen wird.

#### Merke

- Vor einer genetischen Untersuchung (Gentest) muss eine genetische Beratung erfolgen

## Behandlungsoptionen und ethische Aspekte

In Ermangelung einer kausalen Therapie konzentriert sich das derzeitige Management der HK auf die **symptomatische Behandlung**

symptomatische Behandlung.

Die Behandlung von Symptomen im Zusammenhang mit der HK umfasst nicht nur die pharmakologische neurologische und psychiatrische Behandlung, sondern auch nichtpharmakologische Maßnahmen, einschließlich psychotherapeutischer und sozialmedizinischer Begleitung zur Bewältigung psychischer Probleme, Unterstützungsdienste für Menschen mit Behinderungen zur Förderung der Alltagsbewältigung und schließlich Palliativmedizin [6, 12].

Die **maßgeschnittene Behandlung**

maßgeschnittene Behandlung

umfasst ein multidisziplinäres Team (Infobox), das sich sowohl um den Betroffenen selbst als auch um die Aufklärung und Betreuung der Angehörigen kümmert [54]. Das **multidisziplinäre Team**

multidisziplinäre Team

setzt sich aus verschiedenen Berufsgruppen zusammen, die eng zusammenarbeiten. Wichtig ist auch, dass für jeden Betroffenen (inkl. Familie) ein individueller Pflege- und Behandlungsplan erstellt wird.

### Infobox Das multidisziplinäre Team zur Behandlung von Patienten mit Huntington-Krankheit setzt sich aus verschiedenen Berufsgruppen zusammen, die eng zusammenarbeiten

*Ärztliche Versorgung*

- Hausarzt: erste Anlaufstelle, wohnortnah
- Neurologe/Psychiater/Nervenarzt: Diagnostik und Behandlung (pharmakologisch, psychotherapeutisch), nichtmedikamentös (Verordnung von Physio‑, Logo‑, Ergotherapie)
- Humangenetiker: genetische Beratung und molekulargenetische Testung (auch bei Fachärzten mit Qualifikation zur fachgebundenen genetischen Beratung in Huntington-Zentren und Ambulanzen möglich)
- Zahnarzt: Sicherstellung einer adäquaten zahnärztlichen Versorgung
- Weitere Fachärzte je nach Bedarf und Fragestellung (z. B. Palliativmediziner, Internist)

*Psychologische/psychotherapeutische Betreuung*

- Psychologe/Psychotherapeut: psychologische Beurteilung, Beratung und Unterstützung (für Patienten und Angehörige), Psychotherapie
- Neuropsychologe: kognitive Abklärung und Beratung

*Spezifische therapeutische Betreuung*

- Ergotherapeut: Beurteilung der Alltagsfunktionen, kognitives Training, Hilfsmittel
- Logopäde: Beurteilung von Sprache und Kommunikation; Dysphagiediagnostik und -beratung
- Ernährungstherapeut: Beurteilung und Beratung in Ernährungsfragen
- Physiotherapeut: Beurteilung des Gangbildes; Übungsprogramm; Hilfsmittel
- Pflegeberatung/Case-Management: Unterstützung des Patienten und der Angehörigen

*Sozialmedizinische Betreuung*

- Sozialarbeiter: sozialmedizinische Beratung (z. B. Finanzen, Wiedereingliederung, Rente, Pflegestufe, Heimunterbringung, Hilfsmittel)

*Forschung*

- Spezialambulanzen und -zentren: Einbeziehung von Patienten und Angehörigen in die Forschung

*Selbsthilfegruppen*

- Kontakt zu regionalen Vertretern von Selbsthilfegruppen, Peer-to-Peer-Beratung, Kontakt zu Angehörigen und Unterstützung

### Pharmakologische Ansätze

Es gibt es bis heute nur **wenig Evidenz**

wenig Evidenz

aus kontrollierten randomisierten Studien für die Wirksamkeit von Psychopharmaka bei **psychischen Symptomen**

psychischen Symptomen

und Verhaltensstörungen im Rahmen der HK [30]. Die erste Studie dieser Art erbrachte keinen Nachweis der Wirksamkeit von **Bupropion**

Bupropion

bei Apathie durch HK [55]. Obwohl die Behandlung der Chorea nicht Gegenstand dieses Artikels ist, ist es wichtig zu erwähnen, dass eine symptomatische Behandlung Auswirkungen auf die psychiatrische Symptomatik haben kann. Nachdem für **Tiaprid**

Tiaprid,

das bereits seit den 1970er-Jahren zur Behandlung von Bewegungsstörungen (auch bei der HK) eingesetzt wurde, keine heutigen Qualitätsstandards entsprechenden Studien, dafür aber langjährige klinische Erfahrung vorliegen und es außerhalb Europas auch nicht verfügbar ist, empfehlen internationale Leitlinien **Tetrabenazin**

Tetrabenazin

als einziges Medikament, das für die Behandlung der Chorea bei HK zugelassen ist. Tetrabenazin hat aber ein bezüglich psychischer Symptome **ungünstigeres Nebenwirkungsprofil**

ungünstigeres Nebenwirkungsprofil

als Tiaprid. Es kann jedoch laut der Sk2-Leitlinie der Deutschen Gesellschaft für Neurologie [51] erwogen werden, eine antihyperkinetische Therapie zunächst mit Tiaprid einzuleiten und Tetrabenazin (in Kombination oder als Monotherapie) dann einzusetzen, wenn die Behandlungsmöglichkeiten mit Tiaprid (Wirkung, Verträglichkeit) ausgereizt sind [56]. Als Inhibitor des vesikulären Monoamintransporters 2 (VMAT2) reduziert Tetrabenazin die Aufnahme von Monoaminen (einschließlich Dopamin) in die synaptischen Vesikel, was zu Nebenwirkungen im Sinne von Depressionen, suizidalen Gedanken sowie Sedierung führen kann und bei der Verordnung bei HK-Patienten mit affektiven Störungen berücksichtigt werden muss [22, 57].

Zur Behandlung der **motorischen Symptome**

motorischen Symptome

der HK werden **Antipsychotika**

Antipsychotika

wegen ihrer antagonistischen Wirkung auf Dopaminrezeptoren eingesetzt, zeigen aber auch positive Effekte bei gleichzeitigem Vorliegen von Verhaltensauffälligkeiten, Depressionen oder Psychosen [22, 57]. Andere Antipsychotika wie Olanzapin, Risperidon, Sulpirid, Amisulprid, Aripiprazol, Quetiapin, Haloperidol können ebenfalls die Chorea reduzieren [22, 57]. Die Evidenz hierfür stammt jedoch nur aus kleinen, meist offenen Studien und es gibt keine großen direkten Vergleiche zwischen den verschiedenen Medikamenten [58]. Wenn ein Medikament nicht wirkt oder schlecht vertragen wird, ist es sinnvoll, ein anderes Antipsychotikum in Betracht zu ziehen. Als Nebenwirkungen von Antipsychotika können **extrapyramidale Symptome**

extrapyramidale Symptome

(EPS), Herzrhythmusstörungen (Verlängerung der QTc-Zeit) sowie ein metabolisches Syndrom auftreten.

#### Affektive Störungen und Angststörung

Depressionen und Angstzustände treten bei der HK häufig auf. Zur Behandlung können **selektive Serotoninwiederaufnahmehemmer**

selektive Serotoninwiederaufnahmehemmer

(SSRI) wie Sertralin, Escitalopram oder Citalopram und Serotonin-Noradrenalin-Wiederaufnahmehemmer (SNRI) wie Venlafaxin oder Duloxetin eingesetzt werden [22, 59]. Auch Mirtazapin kann hilfreich sein, insbesondere bei Schlafstörungen. Antidepressiva mit ausgeprägten anticholinergen Effekten sollten vermieden werden, da sie choreatische Bewegungsstörungen verstärken können.

Bei **Angststörungen**

Angststörungen

sind Antipsychotika eine wertvolle therapeutische Alternative, wenn andere Behandlungen erfolglos bleiben [12]. Der Einsatz von Benzodiazepinen, z. B. Lorazepam oder Diazepam, sollte zurückhaltend und nur auf der Grundlage einer Risiko-Nutzen-Abwägung in Betracht gezogen werden [45].

#### Antriebsstörung und Apathie

Es ist wichtig, die Angehörigen und das Pflegepersonal über die verschiedenen Aspekte und Ursachen der Apathie aufzuklären [22]. Wenn möglich, werden **aktivierende Maßnahmen**

aktivierende Maßnahmen,

die Einführung von Routinen und ein strukturiertes Programm von Aktivitäten empfohlen. Depressionen können Apathie verstärken. Bei Verdacht auf Depression sollte ein **SSRI**

SSRI

erwogen werden [60]. Bupropion erwies sich in einer randomisierten kontrollierten klinischen Studie ähnlich wie der Alzheimer-Krankheit als nicht wirksam [55]. Sedativa und Antipsychotika können die Apathie verstärken, daher wird empfohlen, bei der Verschreibung darauf zu achten.

#### Zwangsähnliche Verhaltensmuster und Perseveration

Für die Behandlung von Zwangsstörungen sind **SSRI**

SSRI

in der Regel die erste Wahl, wobei auch Komorbiditäten eine Rolle bei der Therapiewahl spielen [22]. Bei mangelndem Ansprechen auf die Therapie wird entweder ein Wechsel auf Clomipramin oder eine Augmentation mit **Stimmungsstabilisatoren**

Stimmungsstabilisatoren

und Antipsychotika bevorzugt, während Benzodiazepine häufiger eingesetzt werden, wenn Angststörungen als Komorbidität vorliegen [61]. Eine Kombination aus medikamentöser Therapie (z. B. mit SSRI) und psychotherapeutischen und psychoedukativen Maßnahmen kann möglicherweise bessere Effekte erzielen [12, 37].

#### Reizbarkeit, Impulsivität und Aggressivität

Bei der Behandlung von Impulsivität und Aggression ist es wichtig, die **verhaltenstherapeutischen Maßnahmen**

verhaltenstherapeutischen Maßnahmen

mit Medikamenten zu kombinieren. Bei akuter Erregung, die nicht auf verhaltenstherapeutische Strategien anspricht, sind **Benzodiazepine**

Benzodiazepine

oder Antipsychotika die bevorzugten pharmakologischen Optionen [45]. Bei chronischer Erregung, die durch wiederkehrende und andauernde Beschwerden gekennzeichnet ist oder bei der die Gefahr besteht, dass der Betroffene sich selbst oder anderen Schaden zufügt, kann man entweder ein **Antipsychotikum**

Antipsychotikum

oder ein stimmungsstabilisierendes Antiepileptikum (z. B. Valproat) einsetzen. Reizbarkeit kann auf SSRIs ansprechen, erfordert aber oft höhere Dosen.

#### Psychose

Ein Antipsychotikum ist die erste Wahl bei der pharmakologischen Behandlung von Psychosen bei der HK, wobei **Antipsychotika der zweiten Generation**

Antipsychotika der zweiten Generation

aufgrund ihres besseren Nebenwirkungsprofils in der Regel bevorzugt werden [45, 62]. Bestehen gleichzeitig ausgeprägte choreatische Bewegungsstörungen, kann der Einsatz eines „klassischen“ Antipsychotikums (z. B. Haloperidol) erwogen werden, dessen dopaminrezeptorantagonistische Wirkungen dann gleichzeitig „antihyperkinetisch“ wirken können.

Eine Umstellung sollte erfolgen, wenn die psychotischen Symptome mit dem ersten Medikament nicht ausreichend kontrolliert werden konnten oder Nebenwirkungen aufgetreten sind. Die **Kombination von Antipsychotika**

Kombination von Antipsychotika

wird nicht empfohlen und sollte nur bei schweren Formen der Psychose eingesetzt werden. Clozapin sollte in Erwägung gezogen werden, wenn die psychotischen Symptome nicht ausreichend auf andere Antipsychotika ansprechen, sofern regelmäßige Blutbildkontrollen möglich sind [22]. Die Herausforderung bei der Behandlung besteht darin, die **Nebenwirkungen**

Nebenwirkungen

der Antipsychotika zu erkennen, da sie schwer von Symptomen der HK im Krankheitsverlauf zu unterscheiden sind.

Im Allgemeinen ist es bei der Behandlung psychiatrischer Symptome der HK wichtig, auf **komorbide Erkrankungen**

komorbide Erkrankungen

zu achten, die den Allgemeinzustand des Patienten verschlechtern können, wie Infektionskrankheiten, Stoffwechselerkrankungen, Drogenkonsum oder andere Ursachen, die zu akuter Agitation oder psychotischen oder deliranten Zuständen führen können. Ebenso ist es wichtig, auf **Schlafhygiene**

Schlafhygiene

als Präventionsstrategie gegen Agitation zu achten und Schlafstörungen zu vermeiden [22].

Bei der Pflege von HK-Patienten im fortgeschrittenen Stadium sollte eine **reizarme Umgebung**

reizarme Umgebung

hergestellt werden. Die Erkrankten profitieren von einem **geregelten Tagesablauf**

geregelten Tagesablauf

und Routinen. Es ist wichtig, die Angehörigen und das Pflegepersonal über die Verhaltenssymptome aufzuklären und sie über Strategien im Umgang mit Erregungszuständen, Perseveration, Apathie und auch über aktivierende, ressourcenorientierte Pflege zu informieren ([54]. Die pharmakologischen Behandlungsmöglichkeiten der Huntington-Krankheit sind in Tab. 1 dargestellt.

**Tab. 1:** Pharmakologische Behandlungsmöglichkeiten bei der Huntington-Krankheit^a^

Symptom	Medikament	Gruppe	Startdosis	Empfehlungsdosis (allg.)
*Affektive Störungen/Angst*	Sertralin	Antidepressivum (SSRI)	25 mg	75–150 mg
	Escitalopram	Antidepressivum (SSRI)	5 mg	10–20 mg
	Citalopram	Antidepressivum (SSRI)	10 mg	20–40 mg
	Venlafaxin	Antidepressivum (SSNRI)	37,5–75 mg	150–225 mg
	Duloxetin	Antidepressivum (SSNRI)	30 mg	60–90 mg
	Mirtazapin	Antidepressivum (NaSSA)	7,5–15 mg	30 mg
	Bupropion	Antidepressivum (SNDRI)	150 mg	300 mg
	Olanzapin	Antipsychotikum 2. Gen	2,5–5 mg	10–20 mg
	Risperidon	Antipsychotikum 2. Gen	0,5–1 mg	2–4 mg
*Zwänge/Perseveration*	Sertralin	Antidepressivum (SSRI)	25–50 mg	75–150 mg
	Paroxetin	Antidepressivum (SSRI)	10 mg	20–40 mg
	Clomipramin	Antidepressivum (TCA)	50 mg	100–150 mg
	Olanzapin	Antipsychotikum 2. Gen	2,5–5 mg	10–20 mg
*Impulsivität/Aggressivität*	Olanzapin	Antipsychotikum 2. Gen	2,5–5 mg	10–20 mg
	Sulpirid/Amisulpirid	Antipsychotikum 2. Gen	50–100 mg	200–400 mg
	Risperidon	Antipsychotikum 2. Gen	0,5–1 mg	2–4 mg
	Zuclopenthixol	Antipsychotikum 1. Gen	5 mg	10–20 mg
	Haloperidol	Antipsychotikum 1. Gen	0,5–1 mg	2–4 mg
	Valproat	Stimmungsstabilisator	150–300 mg	600–900 mg
	Clonazepam	Benzodiazepine	0,25–0,5 mg	1–2 mg
	Diazepam	Benzodiazepine	2,5–5,0 mg	5–10 mg
	Lorazepam	Benzodiazepine	0,5 mg	1–2 mg
*Psychose*	Olanzapin	Antipsychotikum 2. Gen	2,5–5 mg	10–20 mg
	Quetiapin	Antipsychotikum 2. Gen	25 mg	200–400 mg
	Sulpirid/Amisulpirid	Antipsychotikum 2. Gen	50–100 mg	200–400 mg
	Aripiprazol	Antipsychotikum 3. Gen	2,5–5 mg	10–20 mg
	Risperidon	Antipsychotikum 2. Gen	0,5–1 mg	2–4 mg
	Clozapin	Antipsychotikum 2. Gen	12,5 mg	100–200 mg
	Zuclopenthixol	Antipsychotikum 1. Gen	5 mg	10–20 mg
	Haloperidol	Antipsychotikum 1. Gen	0,5–1 mg	2–4 mg
*Chorea/Hyperkinesien*^*b*^	Tiaprid	Antipsychotikum 1. Gen	50–100 mg	400–600 mg
	Tetrabenazine	Antihyperkinetikum	12,5 mg	25–75 mg

*SSNRI* selektive Serotonin-Noradrenalin-Wiederaufnahmehemmer, *SSRI* selektive Serotoninwiederaufnahmehemmer, *NaSSA* noraadrenerge und spezifisch serotonerge Antidepressiva, *SNDRI* selektive Noradrenalin-Dopamin-Wiederaufnahmehemmer, *TCA* trizyklische Antidepressiva, *Gen* Generation

^a^Es handelt sich um Empfehlungen, die auf klinischer Erfahrung, Expertenmeinungen und Leitlinien basieren [12, 22, 57, 59, 62]. Die Dosierungen sollten individuell angepasst und evaluiert werden, eine regelmäßige Überprüfung ist erforderlich

^b^Die antichoreatische Medikation ist nicht Hauptbestandteil des Artikels, daher werden hier nur die am häufigsten verordneten Medikamente zur Übersicht aufgeführt

### Psychotherapie

Psychotherapie (PT) hat sich bei einer Vielzahl HK-bezogener psychischer und Verhaltensproblemen als wertvoll erwiesen. Beispiele hierfür sind die **Unterstützung**

Unterstützung

bei der Entscheidungsfindung vor einer genetischen Untersuchung, die Unterstützung nach einer Befund- oder Diagnosemitteilung, die Krankheitsbewältigung und die Behandlung psychiatrischer Symptome wie z. B. Depression, Angst und Zwang, solange die **kognitiven Ressourcen**

kognitiven Ressourcen

ausreichend sind [63, 64]. Neben einer Symptomlinderung steht die **Verbesserung der Lebensqualität**

Verbesserung der Lebensqualität

im Vordergrund. Auch **Familienangehörige**

Familienangehörige

sind oft stark belastet oder zeigen sogar krankheitsrelevante Symptome, weshalb auch sie von einer PT profitieren können. Da die Pflege von HK-Patienten eine hohe Belastung darstellt, ist es wichtig, auch ihnen Unterstützung anzubieten.

Trotz des Potenzials von PT bei der HK gibt es jedoch nur begrenzte wissenschaftlich fundierte Daten hierzu. Die ohnehin bestehende Komplexität der PT-Forschung wird durch die gleichzeitige Präsenz meist mehrerer psychiatrischer Symptomkomplexe und die Vielfalt der verfügbaren Therapieverfahren bei der HK noch verstärkt, was es schwierig macht, qualitativ hochwertige Wirksamkeitsnachweise zu erbringen. Einige kleine Studien haben einen potenziellen Nutzen gezeigt, z. B. die **Mindfulness-Based Cognitive Therapy**

Mindfulness-Based Cognitive Therapy

(MBCT) für prämanifeste Mutationsträger [64]. Die British Psychological Society hat 2021 Therapieempfehlungen herausgegeben, die für die HK insbesondere **kognitive Verhaltenstherapie**

kognitive Verhaltenstherapie

(Cognitive Behavioral Therapy, CBT), MBCT und **Akzeptanz-und-Commitment-Therapie**

Akzeptanz-und-Commitment-Therapie

(ACT) empfehlen [65].

Bei der HK müssen wegen der fortschreitenden Natur der Erkrankung u. U. die Therapieansätze angepasst werden, um den veränderten Bedürfnissen und Fähigkeiten der Betroffenen gerecht werden zu können. Die Therapieempfehlungen für psychiatrische Symptome bei der HK (z. B. auch in den deutschen Leitlinien) konzentrieren sich häufig auf medikamentöse Ansätze und erwähnen Psychotherapie nur unter „Weitere nicht medikamentöse Therapieoptionen“, wobei angenommen werden kann, dass der Grund hierfür **fehlende wissenschaftliche Wirksamkeitsnachweise**

fehlende wissenschaftliche Wirksamkeitsnachweise

bei der HK sind. In einigen Fällen wird auch eine „psychologische“ oder „psychosoziale Beratung“ empfohlen, wobei es jedoch schwierig sein kann, zwischen Beratungsbedarf und einer behandlungsbedürftigen psychischen Störung zu unterscheiden. Aspekte wie Verfügbarkeit, Finanzierung und versicherungsrechtliche Fragen könnten ebenfalls eine Rolle spielen. Die PT-Forschung entwickelt inzwischen neue Ansätze für eine evidenz- und prozessbasierte modulare Psychotherapie, die in Zukunft auch für die Versorgung von Patienten mit einer HK hilfreich sein könnten. Psychotherapeutische Interventionen können sowohl in Einzel- als auch in Paarsitzungen sinnvoll sein. In den Niederlanden wurde z. B. ein Programm für Paare (Hold Me Tight Program) entwickelt und wissenschaftlich mit 15 Paaren begleitet [66].

### Nichtpharmakologische Ansätze

Physiotherapie, Ergotherapie und Logopädie gehören in der Behandlung der HK mittlerweile zum etablierten Standard, insbesondere zur Verbesserung und Stabilisierung zahlreicher neurologisch-motorischer Symptome [54]. Neben diesen unmittelbaren Wirkungen sind in vielen Studien auch positive Wirkungen auf kognitive Defizite und psychische Symptomkomplexe nachgewiesen worden [67] Zudem helfen regelmäßige ambulante Termine auch bei der **Tagesstrukturierung**

Tagesstrukturierung

und können manchmal auch bei der Überwindung der sonst medikamentös schwer beeinflussbaren Apathie helfen. Bei HK-Betroffenen kommt es regelmäßig zu einer Reduktion der **sozialen Kontakte**

sozialen Kontakte,

sodass auch hier diese unterstützenden Behandlungen zu einer Verbesserung führen können, weil die Therapiekontakte wesentliche Sozialkontakte bedeuten können. Die Entscheidung sollte jedoch individuell und in sorgfältiger Absprache und unter Berücksichtigung der Motivation erfolgen, da diese Therapieverfahren die Betroffenen auch mit den bestehenden Symptomen, Einschränkungen und Defiziten konfrontieren und im Einzelfall dadurch auch zu einer Verschlechterung von Ängsten und depressiven Symptomen führen können [67].

### Ethische Aspekte

Bei der HK gibt es sowohl für Betroffene als auch für Angehörige zahlreiche ethisch-moralische Aspekte, die zu beachten sind. Diese umfassen beispielsweise sowohl subjektive als auch institutionelle ethische Herausforderungen im Zusammenhang mit einer **genetischen Untersuchung**

genetischen Untersuchung.

Beispiele hierfür sind die präzise Aussage zu einer Mutationsträgerschaft bei fehlender kausaler Therapieoption oder Situationen, in denen sich ein (volljähriges) Kind genetisch untersuchen lassen möchte, der mutmaßlich betroffene Elternteil jedoch nicht. In solchen Fällen könnte die **Mutationsträgerschaft**

Mutationsträgerschaft

des Kindes einen unerwünschten Rückschluss auf den Genstatus des Elternteils zulassen. Ein weiteres Dilemma kann bei **eineiigen Zwillingen**

eineiigen Zwillingen

auftreten, wenn nur einer von ihnen eine genetische Untersuchung durchführen lassen möchte. Die Möglichkeit einer prädiktiven genetischen Untersuchung kann schwerwiegende psychologische Auswirkungen haben und Fragen zu Autonomie, Verantwortlichkeit, Fürsorge, Respekt vor den Wünschen anderer und Stigmatisierung aufwerfen.

Weitere ethische Fragen ergeben sich im Zusammenhang mit **Kinderwunsch**

Kinderwunsch

und der Option einer Präimplantationsdiagnostik (PID) oder Pränataldiagnostik (PND). Darüber hinaus bestehen relevante Themen im Zusammenhang mit **lebensverlängernden Maßnahmen**

lebensverlängernden Maßnahmen,

wie die Verwendung einer Ernährungssonde oder Trachealkanüle, Fragen zu Therapielimitationen und einer Palliativversorgung. Die frühzeitige Abfassung von Patientenverfügungen ist zu empfehlen (möglichst gemeinsam mit den behandelnden Ärzten) mit konkreten Festlegungen (z. B. ob und wann – wenn gewünscht – die Anlage einer perkutanen endoskopischen Gastrostomie [PEG] erfolgen soll).

### Palliative Aspekte

Um eine angemessene Palliativversorgung zu gewährleisten, müssen sowohl allgemeine als auch HK-spezifische Symptome und Probleme berücksichtigt werden [68]. Eine bedarfsgerechte Palliativversorgung sollte rechtzeitig eingeleitet werden. Die **häufigsten Todesursachen**

häufigsten Todesursachen

sind rezidivierende Aspirationspneumonien, Herz-Kreislauf-Erkrankungen und Kachexie. Die HK geht mit einer hohen psychischen Belastung einher, z. B. Suizidgedanken, Hoffnungslosigkeit, aber auch Sorge um den Tod und das Sterben [69].

Die **„Angst vor dem Sterben“**

„Angst vor dem Sterben“

stellt eine große Herausforderung dar, wobei die Betroffenen explizit über Angst vor dem Sterbeprozess, Grübeln über den Tod und Angst vor der Zukunft und den Auswirkungen der Erkrankung darauf berichten [69]. Es wird daher empfohlen, psychologische, psychotherapeutische und pharmakologische Maßnahmen zu kombinieren und in die neurologische oder psychiatrische Palliativversorgung einzubeziehen.

Weitere Probleme, die es zu berücksichtigen gilt, sind Schluckstörungen, Kommunikationsschwierigkeiten, Dystonie, Schmerzen sowie kognitive und psychiatrische Symptome [68].

#### Merke

- Das multidisziplinäre Team spielt eine wichtige Rolle bei der Betreuung und Behandlung von HK-Patienten, deren Angehörigen und Betreuern.

#### Wichtige Links.

- Netzwerk für Ärzte, Therapeuten, Forscher und Patienten: European Huntington’s Disease Network (EHDN) https://ehdn.org/
- Deutsche Patientenorganisation: Deutsche Huntington-Hilfe e. V. (DHH) https://www.dhh-ev.de/
- Europäische Patientenorganisation: European Huntington Assoziation (EHA) https://eurohuntington.org/
- Neuigkeiten aus der Huntington-Forschung: HD Buzz https://de.hdbuzz.net/

## Fazit für die Praxis

- Psychiatrische Symptome sind bei der Huntington-Krankheit (HK) häufig und vielfältig. Sie können viele Jahre vor ersten motorischen Symptomen auftreten.
- Bei der HK sind Depressivität und Suizidalität besonders zu beachten.
- Bei fortschreitender Erkrankung können Apathie, aggressive Verhaltensweisen und psychotische Symptome zu gravierenden Problemen führen, die neben einer stationären Behandlung auch eine Heimunterbringung erforderlich machen können.
- In Ermangelung eines kausalen Therapieansatzes erfolgt die Behandlung der psychiatrischen Symptome symptomatisch. Es werden pharmakologische Ansätze, Psychotherapie und weitere nichtpharmakologische Maßnahmen inkl. sozialmedizinische Begleitung eingesetzt. Die pharmakologische und psychotherapeutische Behandlung erfolgt nach den Leitlinien für die Behandlung der jeweiligen Störungsbilder, wobei einige Besonderheiten der HK zu berücksichtigen sind.
- Die Behandlung sollte durch ein multidisziplinäres Team erfolgen und die Beratung der Familienangehörigen einschließen.

## CME-Fragebogen

Was könnte der 38-jährigen Patientin aus dem Fallbeispiel am Anfang des Artikels gegen die Reizbarkeit und das Aufbrausen, was zu vielen Problemen in der Familie geführt hat, empfohlen werden?

Ketamininfusionen im Rahmen eines stationären Aufenthaltes

Methylphenidat und Mediation

MAO(Monoaminoxidase)-Hemmer und systemische Psychotherapie

Selektive Serotoninwiederaufnahmehemmer (SSRI), ggf. atypisches Neuroleptikum und Psychotherapie

Dopaminagonist und Neurofeedback

Durch eine Trias welcher Symptome zeichnet sich die Huntington-Krankheit aus?

Periphere, zentrale und vegetative Symptome

Neurologische, endokrine und metabolische Symptome

Motorisch-neurologische, kognitive und psychiatrische Symptome

Muskuläre, koordinative und sensible Symptome

Schmerzen, Lähmungen und Fatigue

Die Beteiligung welcher Hirnareale erklärt die Vielfalt und Komplexität der klinischen Symptomatik der Huntington-Krankheit?

Basalganglien, frontaler Kortex, Amygdala und Striatum

Kleinhirn, Pons, Subthalamus und Gyrus cinguli

Hirnstamm, Thalamus, Tegmentum und Corpora mamillaria

Hippokampus, limbisches System, Tectum und visueller Kortex

Corpus callosum, Hypothalamus, Vermis cerebelli und Capsula interna

Ein 62-jähriger Patient mit fortgeschrittener Huntington-Krankheit wird wegen einer Sepsis nach Aspirationspneumonie mit bekannten Schluckstörungen und Kachexie stationär aufgenommen. Es liegt eine Patientenverfügung vor, nach der lebensverlängernde Maßnahmen sowie eine PEG(perkutane endoskopische Gastrostomie)-Anlage nicht gewünscht werden. Welche Maßnahme erscheint am sinnvollsten?

Stabilisierung auf einer Intensivstation

Anlage einer PEG-Sonde

Symptombezogene und palliative Behandlung

Vorübergehende parenterale Ernährung

Vorübergehende transnasale Ernährung

Wie häufig sind Suizidversuche bei der Huntington-Erkrankung?

1–2 %

7–10 %

23–25 %

27–29 %

70–80 %

Welcher Befund sollte bei Huntington-Patienten durch weitere Diagnostik abgeklärt werden, weil er untypisch für den Verlauf ist?

Gewichtsverlust

Neurologisch-motorische Symptome

Nierenfunktionsstörungen

Psychische Symptome

Kognitive Einschränkungen und Demenz

In welche Stadien wird die Huntington-Krankheit klinisch deskriptiv eingeteilt?

Asymptomatisch – prodromal – manifest

Akut und chronisch

Langsam progredient – progredient – schubförmig

Psychiatrisch – neurologisch – neuropsychiatrisch

Neurotisch – psychotisch – motorisch

Welche Symptome werden Jahre vor dem Auftreten erster choreatischer Symptome bei der Huntington-Krankheit beobachtet?

Psychiatrische Symptome

Ataxie

Chronisches Schmerzsyndrom

Elektrolytstörungen

Epileptische Anfälle

In Ihrer Praxis stellt sich ein 24-jähriger Mann mit einer positiven Familienanamnese für die Huntington-Krankheit vor, der weder neurologische noch psychiatrische Symptome zeigt und eine genetische Untersuchung auf das Vorliegen einer Mutation für die Huntington-Krankheit wünscht. Wie sollten Sie vorgehen?

Den Patienten darüber aufklären, dass sich bei positiver Familienanamnese eine genetische Untersuchung erübrigt

Eine umgehende Blutentnahme für die molekulargenetische Diagnostik anbieten

Eine genetische Beratung nach dem Gendiagnostikgesetz bei einem hierfür zertifizierten Arzt empfehlen

Den Patienten informieren, dass eine genetische Untersuchung nur beim Vorliegen neurologischer Symptome sinnvoll ist

Dem Patienten in dieser Situation von einer genetischen Untersuchung abraten

Worauf ist besonders zu achten, wenn bei Patienten eine ausgeprägte Chorea mit Tetrabenazin behandelt wird?

Es können Depressionen und suizidale Gedanken ausgelöst werden.

Der Serumkaliumspiegel kann bedrohlich ansteigen.

Eine hormonelle Kontrazeption kann durch Enzyminduktion beeinträchtigt werden.

Es können psychotische und/oder maniforme Episoden ausgelöst werden.

Der Tetrabenazinspiegel ist regelmäßig zu kontrollieren.
